# The science of rapid start—From the when to the how of antiretroviral initiation

**DOI:** 10.1371/journal.pmed.1002358

**Published:** 2017-07-25

**Authors:** Elvin H. Geng, Diane V. Havlir

**Affiliations:** Division of HIV, Infectious Diseases and Global Medicine, Department of Medicine, Zuckerberg San Francisco General Hospital, University of California, San Francisco, California, United States of America

## Abstract

In this Perspective linked to Koenig and colleagues, Elvin Geng and Diane Havlir discuss the next challenges for implementation research around rapid start antiretroviral treatment.

Starting antiretroviral therapy (ART) as soon as possible after HIV infection has clear biological and clinical benefits [1]. HIV devastates the enteric immune system within days of infection [2], creates chronic inflammation [3], and immunological vulnerabilities to infections such as tuberculosis and bacterial pneumonias emerge even when CD4 levels are high [4]. Treatment halts, but cannot fully reverse, this damage. In patients with advanced immunosuppression, accelerating treatment by weeks or days can be lifesaving [5,6]. Successful treatment can also virtually eliminate transmission [7,8]. Medications continue to become less toxic and more convenient. Clinically, sooner is better.

In practice, however, biology depends on behavior. Health systems, healthcare workers, and patients interact to deliver, prescribe, take up, and adhere to medications. HIV diagnosis is a vulnerable time when patients are navigating complicated psychological and social terrains of stigma, threatened relationships, and complex livelihood demands. Offering treatment effectively at this moment requires health systems that are nimble and sensitive. Surprisingly, although studies have found that the introduction of technologies and streamlined clinical operations can minimize delays [9], data are lacking on how to shape what the diagnosis, and the prospect of treatment, means to patients, despite the fact that this meaning is likely to drive subsequent engagement, stabilization, and behavior after HIV diagnosis. Similarly, few data exist for shaping the meaning of illness after diagnoses for other disease conditions (e.g., cancer, diabetes) even though this is of critical importance.

The research article by Koenig and colleagues [10] is a critical step toward addressing the knowledge gap about how fast to start ART. Nonpregnant patients who were newly diagnosed with HIV in voluntary testing and counseling were randomized to same-day initiation of ART or initiation after 21 days. Despite an unselected population, only 7 of 821 patients willing to participate were deemed not ready to be randomized to immediate ART through use of a standardized questionnaire, suggesting that many of today’s patients perceive treatment with, as the authors suggest, a sense of hope that facilitates treatment initiation. Same-day initiation (compared to starting 21 days later) demonstrated clear benefits in this study: a combined outcome of 12-month retention and viral suppression was 44% in the control but 53% in the intervention, while deaths were 6% and 3%, respectively—both statistically significant.

These results extend previous findings. In an individual randomized trial, Rosen and colleagues found rapid ART initiation in South Africa led to a higher proportion of patients initiating ART within 90 days and suppressed at 10 months (51% versus 64%, risk difference: 13%; 95% CI 3% to 23%). In a cluster randomized trial, Amanyire and colleagues showed a multicomponent intervention targeting healthcare workers led to a higher proportion of patients initiating treatment on the same day as clinical eligibility (18% versus 71%, risk difference: 42%; 95% CI 40% to 44%), a higher total fraction on treatment 90 days later, and a higher probability of suppressed viral load 1 year later in an analysis excluding missing values [11]. Although not immediately after diagnosis, Bor and colleagues used an instrumental variable approach to suggest that the act of initiating ART itself improved retention by as much as 70% (95%, CI 42% to 98%) in a real-world cohort in South Africa [12]. In total, these studies provide strong evidence that “first generation” practices of multiple pretreatment counseling sessions to “ensure readiness,” which lead to unintended loss to follow-up and clinical disease progression, should be systematically revised.

The next steps to advancing practice in routine care settings, however, require additional considerations about the study design. Like many trials, Koenig and colleagues’ sought to control the context, which makes application into routine practice settings, in which unintended consequences are the rule, less certain and more indirect [13]. Specifically, the study intervened in more ways than simply same-day treatment initiation. The treatment group also received an accelerated counseling protocol, intensified early visit schedule (4 follow-up visits over 24 days), and both groups received a conditional cash transfer for attendance (which makes frequent counseling visits in the intervention possible). The trial environment exerts a potentially important influence on patient behavior. We cannot automatically assume that the effect of same-day start would be unchanged outside of this context in routine practice settings.

Bringing context into the equation—conceiving of same-day start as an “approach” rather than the isolated act of taking drugs—takes us from the “when” to the “how” of ART start. In practice, same-day start is an outcome, or the result, of interaction between patients, communities, and systems and healthcare workers and not an intervention in and of itself. These interactions are the underlying common causes of both faster start and better retention and also may modify the effect of same-day start on retention and suppression. Patients must desire treatment, invest in it, and become educated and activated. Systems and health care workers must catalyze disclosure when appropriate, enable access (perhaps through differentiated service delivery models), educate, engage patients, and ensure quality—all within a frail infrastructure.

Optimizing how to approach treatment in a way that enables same-day start is more important than ever given global ambitions to treat all persons living with HIV. How we conduct treatment initiation could ultimately play a crucial role in determining epidemic trajectory. The fact that retention and suppression were only 53% with the “winning” approach in the study by Koenig and colleagues, is sobering and underscores that there is great room for improvement. What treatment means to people is a crucial factor in subsequent adherence and retention, and the moments of diagnosis and treatment initiation offer critical windows in which the design of delivery can shape the long-term meaning of treatment. Treatment should not mean the prospect of endless, burdensome facility visits, fear of uncertain side effects with unclear recourse, nor humiliating interactions with healthcare workers. Treatment should mean freedom from fear of disease progression, diminished risk to partners, and a link to a healthcare workforce that can answer questions, help solve problems, and engage patients. How can we change the perception of starting ART that both providers and patients have from burdensome to liberating? How can the critical elements of shaping the meaning of treatment in counseling be identified, standardized, optimized, and consistently delivered? How can health systems, given well-known constraints, act to simultaneously support retention and same-day ART initiation? How do these differ in populations such as pregnant women and adolescents? The answers to these questions can help ensure that treatment is a part of the psychological, spiritual, and human response to HIV as well as the biological one.

Koenig and colleagues’ findings suggest that same-day treatment initiation is superior to the current standard in controlled conditions. These findings imply health systems should seek to offer ART as soon as possible, including on the same day as diagnosis, with adequate education and support provided to those who need it. At the same time, we must invest in learning as we implement—using principles from implementation science—in order to refine the context and shape delivery strategies to guide and support patients through uncertainties of the moment. The initial poor retention of pregnant women starting life-long treatment at diagnosis under option B+ in some settings offers a cautionary tale: when treatment is started cannot be isolated from how it is started [14]. Bringing the “how” into scientific focus is needed to secure the potential benefits of rapid, same-day ART start.
